# Supplementary material for: Multiple-Traits Selection in White Guinea Yam (Dioscorea rotundata) Genotypes
Source: Plants (Basel). 2022 Nov 7;11(21):3003. doi: 10.3390/plants11213003 (PMC9658037; doi:10.3390/plants11213003)
Supplement: Supplementary file 1 [file plants-11-03003-s001.zip › plants-1929539-supplementary.pdf]

SUPPLEMENTARY MATERIALS

Table S1. Eigenvectors, eigenvalues, percent variation and cumulative percent variance of phenotypic breeding values of traits measured in 36 yam genotypes

Traits	PC1	PC2	PC3	PC4	PC5	PC6
Leaf chlorophyll content	0.18	0.24	-0.20	0.41	-0.21	0.40
Stem number per plant	0.19	0.27	-0.21	0.37	-0.25	0.32
Number of tubers per plant	0.10	0.13	0.53	-0.09	-0.08	0.03
Tuber yield (t ha^{-1})	0.18	-0.08	-0.13	-0.26	-0.56	-0.16
Ash content (%)	-0.04	-0.13	0.37	-0.11	-0.44	0.11
Breakdown value (cP)	0.28	-0.34	0.06	0.10	0.11	0.09
Dry matter content (%)	0.33	0.12	0.18	0.12	0.15	-0.24
Final paste viscosity (cP)	-0.04	0.46	0.19	0.07	0.06	-0.20
Flour yield (%)	0.30	0.13	-0.10	-0.41	0.10	0.16
Holding strength (cP)	-0.22	0.39	0.12	-0.03	-0.08	-0.23
Peel loss (%)	-0.12	-0.21	0.12	0.51	-0.18	-0.38
Protein content (%)	-0.16	-0.31	0.19	-0.09	-0.06	0.43
Pasting temperature (°C)	-0.34	0.05	-0.16	-0.15	0.15	0.18
Pasting time (s)	-0.21	0.38	0.00	-0.17	-0.21	0.13
Peak viscosity (cP)	0.36	-0.03	0.19	0.12	0.11	0.06
Starch yield (%)	0.36	0.15	0.02	-0.14	0.28	0.00
Yam mosaic virus	-0.31	0.01	0.07	0.23	0.36	0.17
Stem diameter (mm)	0.04	-0.09	-0.52	-0.06	-0.04	-0.31
Eigenvalue	5.15	3.30	2.41	1.70	1.62	0.96
% of variance	28.59	18.34	13.39	9.72	8.98	5.32
Cumulative % of variance	28.59	46.93	60.32	70.05	79.03	84.35

PC=principal component

Table S2. BLUP values for 18 agronomic traits measured in 36 genotypes of white yam

Genotype	YMV	PTEMP	PTIME	PLOSS	SCMR	STNP	STDP	TBRNP	Yield
TDr0000362	1603.50	81.95	5.30	22.55	45.06	2.48	6.12	2.31	9.71
TDr04-219	1187.28	82.74	5.37	17.20	40.96	1.65	5.87	1.97	6.35
TDr06-15	1135.14	83.62	5.04	27.60	43.06	1.27	6.36	1.34	4.88
TDr08-21-2	965.91	81.12	5.06	22.75	46.34	1.76	6.31	1.73	19.33
TDr08-21-3	1141.48	83.58	4.94	31.85	40.50	1.43	6.26	1.34	13.87
TDr8902157	1052.83	81.74	5.12	20.65	44.47	1.49	6.70	1.58	11.01
TDr8902475	530.79	80.53	5.12	37.50	47.23	2.09	6.48	2.08	23.06
TDr8902607	936.28	83.40	5.36	18.90	47.29	1.10	6.85	1.18	10.85
TDr8902665	908.28	80.74	5.02	24.40	44.20	2.81	6.03	2.26	7.85
TDr8902677	1314.66	82.15	5.77	33.05	44.55	1.93	6.05	2.18	8.69
TDr9501932	871.14	82.12	4.95	24.80	44.33	1.49	6.89	1.44	19.49
TDr9518544	893.51	81.12	5.00	25.70	44.21	3.86	5.78	3.11	19.18
TDr9518988	807.78	80.53	5.04	20.20	44.63	1.82	6.64	1.63	11.65
TDr9519156	1406.91	81.57	4.98	27.60	43.10	1.82	6.32	1.81	12.12
TDr9519158	896.30	82.39	5.59	18.20	43.48	1.49	6.79	1.60	18.02
TDr9519177	1200.59	81.74	4.90	34.35	43.20	1.43	6.60	1.18	15.86
TDr9600629	1435.79	83.19	5.06	33.50	45.36	1.32	6.37	1.34	10.75
TDr9700205	1219.62	83.16	5.36	21.60	44.58	1.27	6.75	1.50	10.90
TDr9700632	1445.11	83.63	5.20	26.35	46.00	1.10	6.79	1.15	11.94
TDr9700793	1054.70	82.34	4.92	33.75	42.85	1.38	6.71	1.26	17.76
TDr9700917	1116.73	84.20	6.06	16.50	44.28	1.60	6.89	1.73	14.42
TDr9902562	900.56	82.11	4.95	36.90	44.72	1.38	6.75	1.26	15.09
TDr9902626	1463.74	82.33	4.96	34.20	47.54	1.71	6.37	1.87	12.82
TDr9902789	1430.81	83.38	5.40	47.40	43.78	1.19	6.71	1.26	14.75
TDrAgbanwobe	1444.17	82.52	5.63	21.75	44.18	1.21	6.62	1.26	8.20
TDrAlumaco	964.32	81.72	5.44	29.80	49.08	1.27	6.24	1.44	12.44
TDrEhobia	1159.06	82.20	5.30	25.75	44.90	1.76	5.99	2.00	12.91
TDrFakesta	875.80	80.92	5.00	16.10	48.68	1.60	6.89	2.08	17.69
TDrLeusi	780.36	83.24	5.18	17.25	45.41	1.21	6.54	1.68	19.57
TDrMeccakusa	699.70	82.40	5.04	30.30	45.18	1.16	7.30	1.29	15.65
TDrNndu	718.33	82.61	5.55	18.20	45.55	1.32	6.40	1.13	23.12
TDrOjuiyawo	1008.42	82.58	5.12	25.30	45.25	1.38	7.20	1.42	15.17
TDrPampars	807.78	81.91	4.96	18.30	46.79	1.16	6.87	1.18	16.81
TDrPouna	1113.40	81.92	5.08	15.85	45.25	1.65	6.50	1.52	15.51
TDrUfenyi	1333.29	83.04	4.98	20.15	43.01	1.65	6.93	1.58	12.23
TDrYangbedu	1006.20	82.55	4.90	26.60	43.76	1.21	7.25	1.26	10.23

YMV: yam mosaic virus; PTEMP: pasting temperature; PTIME: pasting time; PLOSS: peel loss; SCMR: leaf chlorophyll content; STNP: stem number per plant; STDP: stem diameter per plant; TBRNP: tuber number per plant; Yield: tuber yield;

Table S2. Continued.

Geno	DMC	PV	HS	BV	FPV	SYLD	FYLD	Protein	ASHC
TDr0000362	32.11	2497.49	1771.22	332.81	3881.55	17.21	24.00	4.26	2.43
TDr04-219	37.16	2636.78	1416.36	290.39	3764.99	25.23	26.14	3.47	2.45
TDr06-15	30.57	2228.10	1096.44	344.19	2958.63	14.95	19.87	4.31	2.62
TDr08-21-2	33.62	2909.31	726.08	443.50	3291.38	18.76	28.13	4.42	2.79
TDr9700793	28.66	2160.03	658.02	407.55	2420.87	12.56	21.37	5.05	2.56
TDr8902475	35.04	2525.49	1028.99	438.33	3134.97	19.07	29.93	4.10	2.34
TDr9700632	37.18	2741.30	644.05	487.73	3250.16	17.03	25.41	4.16	2.64
TDr9700205	32.39	2195.75	1676.13	275.65	3515.22	16.99	25.99	4.73	2.62
TDr08-21-3	38.30	2900.62	707.85	787.11	3043.80	21.37	28.76	4.47	2.43
TDr9519158	30.17	2048.50	1725.95	147.88	3528.32	13.54	18.99	4.11	2.89
TDr9518988	34.27	2266.00	884.98	499.37	2647.71	19.60	26.38	4.37	3.03
TDr8902157	34.68	2627.60	561.11	652.23	2604.31	18.54	26.48	4.69	3.56
TDr8902665	35.93	2586.32	802.34	474.28	2962.18	21.29	30.22	4.22	2.45
TDr8902677	33.88	2223.75	723.65	389.96	2570.46	17.65	26.72	4.00	2.72
TDr9501932	31.15	2238.00	1649.39	228.83	3588.38	16.06	28.66	3.90	2.61
TDr9518544	29.32	2365.21	772.56	496.78	2661.63	15.00	23.03	4.53	2.67
TDr9519156	33.29	2013.74	946.05	257.80	2672.28	16.19	26.86	4.37	2.52
TDr9519177	30.16	2221.58	915.66	334.87	2798.67	15.79	24.73	3.95	2.49
TDr9600629	28.60	2275.65	751.90	369.01	2676.65	15.26	25.55	4.32	2.35
TDr9700917	30.00	2248.62	1026.56	429.80	2828.69	14.60	20.79	4.05	2.68
TDr9902562	27.85	2005.78	1369.88	170.64	3136.06	14.33	24.82	4.37	2.74
TDr8902607	32.93	2227.13	798.09	402.12	2628.33	13.98	23.07	4.26	2.50
TDr9902626	32.74	2403.59	763.75	436.78	2760.45	15.17	20.89	4.21	2.55
TDr9902789	33.65	2246.93	1617.49	343.67	3448.61	12.52	17.49	3.89	2.83
TDrAgbanwobe	27.66	2134.68	1038.71	201.16	2952.08	14.20	22.93	4.95	2.56
TDrAlumaco	34.63	2385.24	749.17	299.70	2871.28	18.19	24.24	3.36	2.80
TDrEhobia	33.40	2168.47	1444.92	299.96	3250.98	16.15	28.18	4.47	2.91
TDrFakesta	33.02	2461.77	958.20	419.71	3018.95	21.15	28.18	3.95	2.30
TDrLeusi	32.65	2166.06	752.21	351.17	2571.82	18.58	30.02	3.94	2.39
TDrMeccakusa	30.71	2469.25	693.57	518.25	2685.65	16.02	25.94	4.37	2.48
TDrNndu	34.68	2229.06	1631.77	185.12	3608.58	18.27	31.04	4.21	2.71
TDrOjuiyawo	34.32	2392.25	682.03	416.86	2695.21	18.85	27.16	3.79	2.45
TDrPampars	32.62	2671.29	657.11	651.97	2740.25	20.27	27.35	4.42	2.73
TDrPouna	32.17	2563.15	649.21	589.38	2676.92	18.67	29.44	3.79	2.30
TDrUfenyi	36.89	2483.97	768.46	508.94	3044.34	20.44	27.06	3.79	2.65
TDrYangbedu	35.68	2437.87	744.31	566.36	2644.98	19.47	27.25	4.48	2.54

DMC: dry matter content; BV: breakdown value; FPV: final paste viscosity; FYLD: flour yield; ASHC: ash content; SYLD: starch yield; PV: peak viscosity; HS: holding strength; Protein: protein content

Table S3. Description of breeding lines utilized for the study

SN	Genotype	Status	Pedigree
1	TDr0000362	Improved	TDr9505576/TDr9800062
2	TDr04-219 (Amula)	Landrace	Unknown
3	TDr06-15	Landrace	Unknown
4	TDr08-21-2 (Ekpe-I)	Landrace	Unknown
5	TDr08-21-3(Ekpe-II)	Landrace	Unknown
6	TDr8902157	Improved	Unknown
7	TDr8902475	Improved	Unknown
8	TDr8902607	Improved	Unknown
9	TDr8902665	Improved	Nsukka/Unknown (OP)
10	TDr8902677	Improved	Unknown
11	TDr9501932	Improved	TDr8600309/Unknown (OP)
12	TDr9518544	Improved	TDr8700571/Unknown (OP)
13	TDr9518988	Improved	TDr8700571/Unknown (OP)
14	TDr9519156	Improved	Unknown
15	TDr9519158	Improved	TDr8700334/Unknown (OP)
16	TDr9519177	Improved	Unknown
17	TDr9600629	Improved	TDr93:1/Unknown (OP)
18	TDr9700205	Improved	TDr8700839/TDr8700552
19	TDr9700632	Improved	TDr93:24/TDr8902494
20	TDr9700793	Improved	TDr93:23/Unknown (OP)
21	TDr9700917	Improved	TDr8901892/IN94R-2
22	TDr9902562	Improved	TDr93:1/TDr9500858
23	TDr9902626	Improved	TDr93:25/TDr9500858
24	TDr9902789	Improved	TDr93:50/TDr9502026
25	Agbanwobe	Landrace	Unknown
26	Alumaco	Landrace	Unknown
27	Ehobia	Landrace	Unknown
28	Fakesta	Landrace	Unknown
29	Leusi	Landrace	Unknown
30	Meccakusa	Landrace	Unknown
31	Nndu	Landrace	Unknown
32	Ojuiyawo	Landrace	Unknown
33	Pampars	Landrace	Unknown
34	Pouna	Landrace	Unknown
35	Ufenyi	Landrace	Unknown
36	Yangbedu	Landrace	Unknown

Table S4. Phenotypic traits measured in 36 white yam genotypes

SN	Trait/ descriptor	Trait acronym	Score code – descriptor state	Sample/time collected
1	Leaf chlorophyll content (SPAD value)	SCMR	direct measurement (measured on 3 fully opened leaves using SPAD Chlorophyll meter reading)	on 8 plants at 5 MAP
2	Stem number per plant	STNP	direct measurement: done by counting	on 8 plants at 5 MAP
3	Stem diameter per plant (cm)	STDP	direct measurement: done using vernier caliper	on 5 plants at 5 MAP
4	Yam mosaic virus severity	YMV	1=no visible symptom of disease; 2=mild; 3=low; 4=intermediate; 5=high	on 8 plants at 60, 75, 90, 105, 120, 135, 165 and 180 DAP
5	Tuber yield (t ha^{-1})	TBRYLD	direct measurement	
6	Number of tubers per plant	TBRNP	direct measurement: done by counting	
7	Dry matter content (%)	DMC	direct measurement	
8	Peel loss (%)	PLOSS	direct measurement	
9	Starch yield (%)	SYLD	direct measurement	
10	Pasting temperature (°C)	PTEMP	direct measurement	
11	Pasting time (s)	PTIME	direct measurement	
12	Peak viscosity (cP)	PV	direct measurement	
13	Holding strength (cP)	HS	direct measurement	
14	Breakdown value (cP)	BV	direct measurement	
15	Final paste viscosity (cP)	FPV	direct measurement	
16	Flour yield (%)	FYLD	direct measurement	
17	Ash content (%)	ASHC	direct measurement	
18	Protein content (%)	PROTEINC	direct measurement	

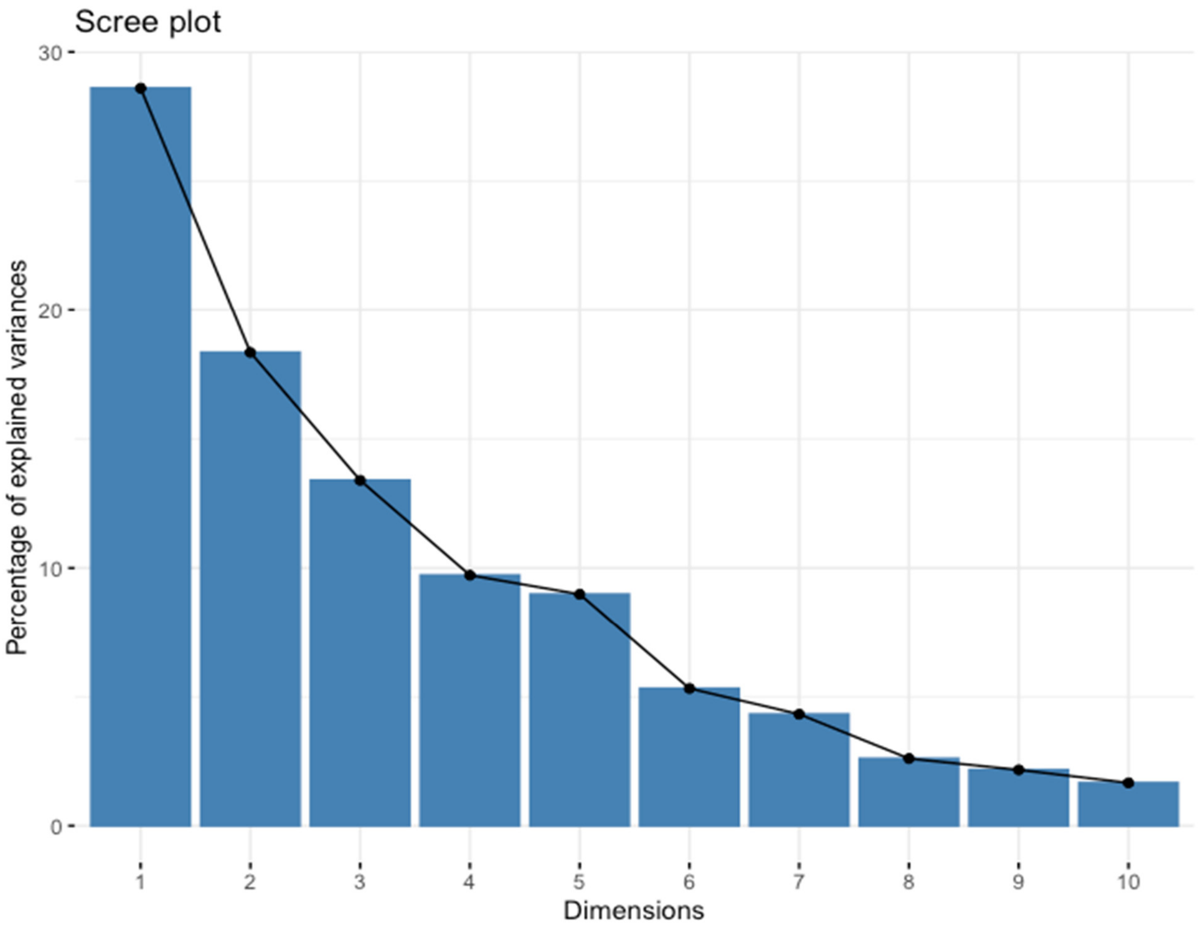

Figure S1. Scree plot of percent variation versus principal components of phenotypic breeding values of traits measured in 36 yam genotypes
